# *Mucispirillum schaedleri*: Biofilm Architecture and Age-Dependent Pleomorphy

**DOI:** 10.3390/microorganisms11092200

**Published:** 2023-08-31

**Authors:** Aléhandra Desjardins, Patricia Zerfas, Dominic Filion, Robert J. Palmer, Emilia Liana Falcone

**Affiliations:** 1 Center for Immunity, Inflammation and Infectious Diseases, Montreal Clinical Research Institute (IRCM), Montreal, QC H2W 1R7, Canada; alehandra.desjardins@ircm.qc.ca; 2 Department of Microbiology, Infectious Diseases and Immunology, Université de Montréal, Montreal, QC H3T 1J4, Canada; 3 Division of Veterinary Resources, Office of Research Services, National Institutes of Health (NIH), Bethesda, MD 20892, USA; 4 Microscopy and Imaging Platform, Montreal Clinical Research Institute (IRCM), Montreal, QC H2W 1R7, Canada; 5 National Institute of Dental and Craniofacial Research, National Institutes of Health (NIH), Bethesda, MD 20892, USA; 6 Department of Medicine, Université de Montréal, Montreal, QC H3T 1J4, Canada

**Keywords:** *Mucispirillum schaedleri*, pleomorphism, biofilm, round body, spirochete, cording, mycobacteria

## Abstract

Round bodies in spirochete cultures have been a controversial subject since their description seven decades ago. We report the existence of round bodies (spherical cells) in cultures of *Mucispirillum schaedleri*, a spiral bacterium phylogenetically distant from spirochetes. Furthermore, when grown in biofilms, *M. schaedleri* demonstrates a unique morphology known as cording, which has been previously described only in mycobacteria. Thus, *M. schaedleri* has two distinct features, each previously thought to be unique to two different phylogenetically distant groups of bacteria.

## 1. Introduction

*Mucispirillum schaedleri* (*Deferribacteraceae*) is a spiral-shaped, flagellated, Gram-negative, obligately anaerobic bacterium that was first described in 1987 as “a spirochete” [1]. Its original designation, Altered Schaedler Flora strain 457 (ASF 457), relates to its inclusion in commercially supplied consortia used to establish putatively standardized bacterial communities in gnotobiotic mice [2,3]. A decade later, unclassified murine gut isolates similar to ASF 457 (but not then recognized as such) were obtained during a thesis project [4]; five had novel 16S rRNA gene sequences most closely related to *Geovibrio* and *Deferribacter*. Shortly thereafter, a molecular phylogenetic study on the Altered Schaedler Flora found the 16S rRNA gene sequence of ASF 457 to be closely related to those of the unclassified murine isolates [5]. Six years on, seven additional murine isolates were obtained that had 16S rRNA gene sequences differing by maximally four nucleotides, and four had 100% similarity to ASF 457. Based on the close phylogenetic relationships and certain phenotypic features, the designation *Mucispirillum schaedleri* was proposed [6]. The type strain (HRI |17) was deposited in the Australian Collection of Microorganisms (ACM 5223) and in the American Type Culture Collection (ATCC BAA-1009), but it is no longer available. Two variants of ASF 457 referred to as AYGZ [7] and MCS [8] have been reported, and a variant named SH1 (likely to be MCS) is available from the German Collection of Microorganisms and Cell Cultures (DMSZ). The strains have few genetic differences [8], and phenotypic differences have yet to be reported. Published microscopy is limited to an electron microscopy micrograph [6] and fluorescent in situ hybridization (FISH) documentation in artificially colonized mice [8,9].

Murine intestinal samples are enriched in *M. schaedleri* in the context of infection [10], as well as in genetic [11,12,13] and chemical [14] colitis models. Intestinal enrichment has also been described in other murine models including stress [15], high-fat diet [16] and rheumatoid arthritis [17]. Importantly, molecular data suggest that *M. schaedleri* colonizes the nasal, vaginal and GI mucosae of humans, albeit at a lower prevalence than in mice or other sources [18]. *M. schaedleri* abundance was found to be increased in a human study of Parkinson’s disease [19]. Enrichment may relate to the ability of *M. schaedleri* to reduce nitrate [8], which is abundant in inflammatory conditions [18]. It harbors only a few genes encoding glycan-degrading enzymes; instead it utilizes monosaccharides, amino acids and short-chain fatty acids [8]. *M. schaedleri* also contains numerous genes associated with nitrate reduction, as well as genes for superoxide reductase, catalase and cytochrome c oxidase genes [8]: features that may facilitate colonization of mucosal surfaces in the presence of epithelium-derived reactive oxygen and nitrogen species [20,21].

Spirochetes, a group of Gram-negative anaerobic motile spiral bacteria unrelated to *M. schaedleri*, can show pleomorphic growth in which spherical bodies are intermixed with the typical spiral morphologies. Multiple terms appear in the literature (cysts, round bodies, spheroids, gemmae), some of which have been proven inapplicable (L-forms, cell-wall-deficient forms); the term round body will be used here. Best studied in the Lyme disease pathogen *Borrelia burgdorferi* and in *Treponema* spp., round bodies contain rod-like cells appressed to the inner membrane of the sphere in a folded or looping manner. Their formation is thought to take place through the development of a bleb in the outer membrane of the typical spiral cell (frequently on the end) into which the cell moves by rolling up to fit within the diameter of the expanding bleb [22]. Electron micrographs show that a large portion of the sphere’s diameter can be occupied by cellular cross sections, and such images can give the impression that multiple cells are sometimes present. Spheres can also contain amorphous material. Formation is reported to be induced by stressors (antibiotics, hypotonic conditions, changes in serum or complement in the medium and changes in oxygen tension) [23,24,25] which favor membrane blebbing and the development of gaps between the outer membrane and cell wall. Round bodies are typically observed in greater numbers in older cultures [23,26]. They have been described as reproductive or resistant forms from which a single cell or multiple cells emerge [27,28], and they have been observed in vivo in a murine infection model [29]. Reviews of the Lyme disease literature have not shown a clear connection between round bodies and disease, but at the same time, they have noted a potential role for pleomorphy in infection and persistence [30,31].

The present study demonstrates that *M. schaedleri* produces round bodies identical to those observed in spirochetes and that *M. schaedleri* exhibits a striking in vitro biofilm morphology which mirrors cording in mycobacteria.

## 2. Materials and Methods

### 2.1. Bacterial Strains and Growth Conditions

*M. schaedleri* ASF 457 (AYGZ variant) was kindly provided by Dr. Michael Wannemuehler (Iowa State University, Ames, IA, USA) and cultured on Akkermansia minimal medium (AMM) broth [8] containing 1% KNO_3_ in a Whitley A35 anaerobic chamber (Microbiology International, Frederick, MD, USA) under N_2_/CO_2_/H_2_ 80/10/10, 37 °C. *Treponema denticola* ATCC 35405 was grown in NOS broth [32] in the anaerobic chamber. Green fluorescent protein (GFP)-expressing *Mycobacterium bovis* BCG [33] was grown in Middlebrook 7H9 base supplemented with 0.2% (*v*/*v*) glycerol at 37 °C in a 3% CO_2_ incubator.

### 2.2. Initial Microscopic Characterization

All manipulations except centrifugation and microscopy were carried out in the anaerobic chamber, and all liquids were equilibrated in the chamber overnight. For initial observation of cells from culture, washed cells were resuspended in BacLight™ Live/Dead viability stain (Invitrogen, Waltham, MA, USA) prepared in anaerobic phosphate-buffered saline (PBS) and examined by confocal microscopy (Leica SP8, Leica Microsystems, Wetzlar, Germany). Excitation wavelength was 488 nm, and emission was collected simultaneously at 505–530 nm (Syto9™—“live”) and 600–630 nm (propidium iodide—“dead”) using a 100× (1.4 NA) oil-immersion lens. Optical section thickness and z-step settings were those recommended by the system software. Transmitted white-light micrographs of wet-mount preparations were obtained using a 100× (1.4 NA) oil-immersion lens on a Leica DM LB2 (Deerfield, IL, USA) microscope with a Hamamatsu (Bridgewater, NJ, USA) Orca camera.

### 2.3. Transmission Electron Microscopy

Cell pellets from broth culture or loopfuls of material from AMM plates were fixed for 48 h at 4 °C in 0.1 M cacodylate buffer (pH 7.4) containing glutaraldehyde/paraformaldehyde (2.5%/1%), then washed 3 times with cacodylate buffer. The pellets were dehydrated in ethanol and propylene oxide, then embedded in EMBed 812 resins (Electron Microscopy Sciences, Hatfield, PA, USA). Thin sections (80 nm) were cut using an Ultracut-UCT ultramicrotome (Leica Biosystems, Wetzlar, Germany), placed onto 300-mesh copper grids, then stained with saturated uranyl acetate in 50% methanol followed by lead citrate. Grids were viewed with a JEM-1200EXII electron microscope (JEOL Ltd., Tokyo, Japan) at 80 kV; images were recorded on a mid-mounted CCD camera (XR611M, 10.5 Mpixel; Advanced Microscopy Techniques Corp., Woburn, MA, USA).

### 2.4. Biofilm Experiments

For *M. schaedleri* biofilms, a 4-day-old broth-culture cell pellet was resuspended in AMM/NO_3_ to 0.05 OD600. Three microliters of the standardized cell suspension were added to wells of duplicate chamber slides (Ibidi Gmbh, Gräfelfing, Germany; µ-Slide 8-well, untreated glass), each containing 300 µL of AMM/NO_3_. After 18 h, one set of slides was processed by removal of spent culture followed by 2 washes with 250 µL of PBS. Two hundred microliters of BacLight™ Live/Dead stain were added to the biofilms, and the slides were incubated for 15 min prior to confocal microscopy as described above. The second set of slides was processed the following day (ca. 48 h incubation). In addition to high-resolution images, six randomly selected fields of view around the center of the chamber were recorded using a 20×, 0.75 NA dry lens. For flowcell-grown biofilms, 2 mL of the standardized suspension were injected into two µ-Slide I 0.8 glass-bottom chambers (Ibidi LLC) connected in series. After 60 min of attachment time, flow of AMM/NO_3_ was begun at 10 mL/h (Ismatec^®^ Reglo Independent Channel Control pump, Cole-Parmer, Vernon Hills, IL, USA). After 18 h, one flowcell was removed and slowly injected with 1 mL of viability stain; the second flowcell was processed after 48 h incubation. High-resolution and low-magnification images were collected as for chamber biofilms. Imaris V9.7 (Oxford Instruments, Abingdon, UK) was used to compute biovolume, to produce maximum-intensity projections and to generate movies. For *Treponema denticola* biofilms, an overnight culture was diluted 10× into 300 µL of fresh NOS in an Ibidi chamber slide. After 18 h of growth in the anaerobic chamber, the biofilm was examined using Live/Dead stain as above. For *Mycobacterium bovis* biofilms, an overnight culture was diluted 10× into 300 µL of fresh medium in an Ibidi chamber slide. After 18 h of growth in the CO_2_ incubator, the pellicle biofilm at the air–liquid interface was lifted off using an inoculating loop, placed on a wetted microscope slide and examined as above but using intracellular GFP fluorescence rather than Live/Dead stain.

### 2.5. Enumeration of Round Bodies by Microscopy

For experiments on temporal aspects of round body production, cell pellets from 3- or 6-day-old ASF 457 broth cultures were pelleted and resuspended in anaerobic PBS with BacLight™ Live/Dead stain. Culture ages investigated here correspond approximately to mid-log (3 days) and plateau (6 days) phases on a growth curve measured by OD [8]. Wet-mounted samples were visualized by confocal fluorescence microscopy using an LSM710 (Carl Zeiss Canada Ltd., North York, ON, Canada) with a 63× (1.4 NA) oil-immersion objective. The excitation/emission wavelengths for “live” (Syto9™) and “dead” (propidium iodide; PI) stains were 488/531 nm and 534/616 nm, respectively. For each experimental condition, 9 scanned images (3048 × 3048 pixels, 269.0 µm × 269.0 µm) were taken and 4 independent experiments were performed (*n* = 4). Raw image data were imported to MATLAB in tiff format. Syto9 and PI tiff images were segmented using 3 labels from the Pixel Classification algorithm from ilastik (version 1.3.3post3; https://www.ilastik.org/download (accessed on 25 August 2023)). Two labels were identified as the Syto9 and PI channels, and the background was set as the third channel. Objects found by segmentation were processed using MATLAB (version 2020b; https://www.mathworks.com (accessed on 25 August 2023)). A first criterion based on size was used to remove all objects smaller than 0.4 µm^2^ (corresponding to 50 pixels). Objects were then selected based on their Eccentricity and Solidity parameters. Threshold values of Eccentricity lower than 0.9 and Solidity greater than 0.7 were used to identify round bodies, whereas objects not corresponding to these criteria were identified as rods. Finally, a mean intensity threshold value of 25 was used to reject faint round objects from the Syto9 channel (Supplementary Figure S1).

### 2.6. Statistical Analyses

For 2-group comparisons, the Mann–Whitney U test was performed using Prism 9 (version 9.5.1), and significance was established at *p* < 0.05.

## 3. Results

### 3.1. M. schaedleri ASF 457 Produces Round Bodies

**Figure 1:**
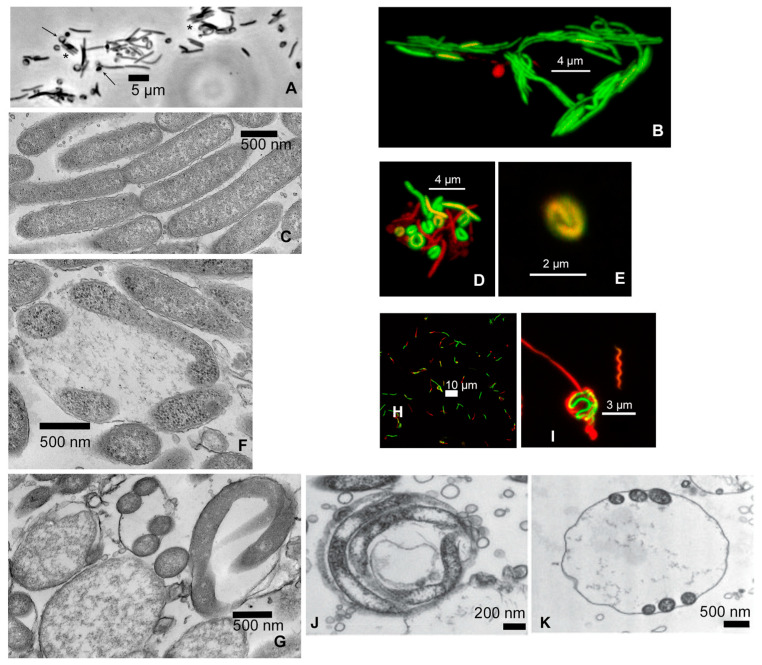
Light and electron microscopy of round bodies from *Mucispirillum schaedleri*, *Treponema denticola* and *Borrellia burgdorferi*. (**A**) *M. schaedleri* ASF 457 from plate culture. Appressed cells (stars) and round bodies attached to tips of rods (arrows). Note the crescent-shaped regions on the walls of round bodies. Transmitted light micrograph, bar = 5 µm. (**B**) Confocal micrograph of viability-stained cells from 3-day-old liquid culture, bar = 4 µm. (**C**) Electron micrograph of appressed cells from liquid culture, bar = 500 nm. (**D**) Confocal micrograph of viability-stained cells from 6-day-old broth culture showing crescent-shaped cells (round bodies); fully active cells (green) together with those having reduced activity (red, orange), bar = 4 µm. (**E**) Confocal micrograph of viability-stained round body from 6-day-old broth culture showing spiral morphology, bar = 2 µm. (**F**,**G**) Electron micrographs of round bodies from broth culture. Crescent- and helical-shaped cellular cross sections are visible within the round bodies (bar = 500 µm). Multiple cross sections are visible in single round bodies, and round bodies also have amorphous contents. (**H**) *Treponema denticola* biofilm showing round bodies (from current study, bar = 10 µm). (**I**) High-magnification micrograph of round body from center of H (bar = 3 µm). (**J**,**K**) Electron micrographs of *Borrellia burgdorferi* from a different study [22].

*M. schaedleri* ASF 457 presented three prominent morphological features in static broth culture. First, rods were frequently found appressed to one another in an arrangement similar to that observed in, e.g., *Capnocytophaga* [34] (Figure 1A–C). Second, round bodies were often attached to the end of rods and also distributed as single entities, in which case they were often larger than when attached to a rod. The round bodies typically contained cells appressed to the inner wall of the sphere, presenting either a c-shaped or spiral appearance (Figure 1A,D,E). Electron microscopy confirmed that the cell inside the round body can be wound along the sphere’s wall, thereby giving the appearance of multiple cells, and that many round bodies had amorphous content (Figure 1F,G).

**Figure 2:**
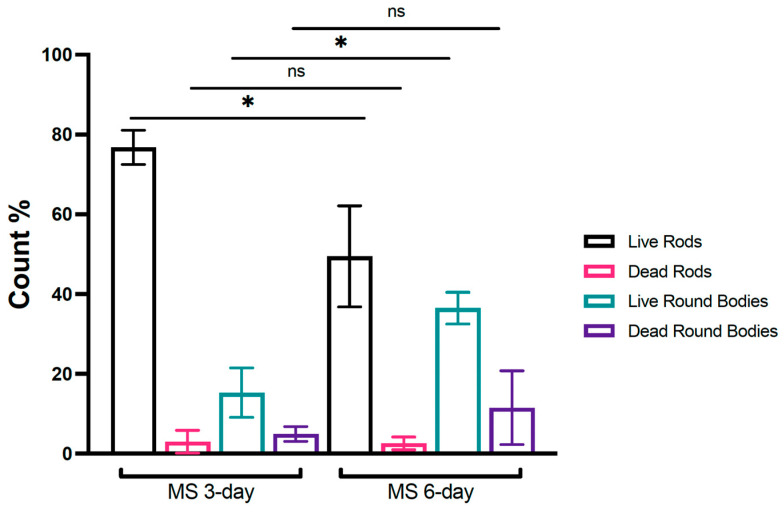
Quantification of rods and round bodies in 3- and 6-day-old *Mucispirillum schaedleri* cultures. MS 3-day and 6-day cultures; *n* = 4 independent experiments. All values are presented as the mean ± standard deviation (SD), * *p* < 0.05 was considered significant. MS, *Mucispirillum schaedleri*; ns, not significant.

For comparison, confocal micrographs of round bodies produced by *Treponema denticola* biofilms grown as part of the current study (Figure 1H,I), and electron micrographs from a different study [35] on *Borrellia burgdorferi* (Figure 1J,K), are shown. Third, image quantification analysis of broth-grown cultures demonstrated that the ratio of “live” round bodies to “live” rods increased as cultures aged (Figure 2 and Supplementary Table S1). In 3-day-old cultures, round bodies made up 20.3% of all cells counted, and 15.3% of the round bodies were “live”. In 6-day-old cultures, 48% of counted cells were identified as round bodies, of which 36.5% were “live”. Regardless of culture age, more than 95% of rods were scored as “live”. Notably, the proportion of “live” rods was significantly decreased at 6 days (49.4%) compared to 3 days (76.8%) (*p* = 0.03), whereas the proportion of “live” round bodies was significantly increased at 6 days (36.5%) compared to 3 days (15.3%) (*p* = 0.03). These data are consistent with previously described age-associated formation of round bodies in spirochete cultures [36,37].

### 3.2. M. schaedleri ASF 457 Biofilms Are Composed of Cords and Contain Round Bodies

**Figure 3:**
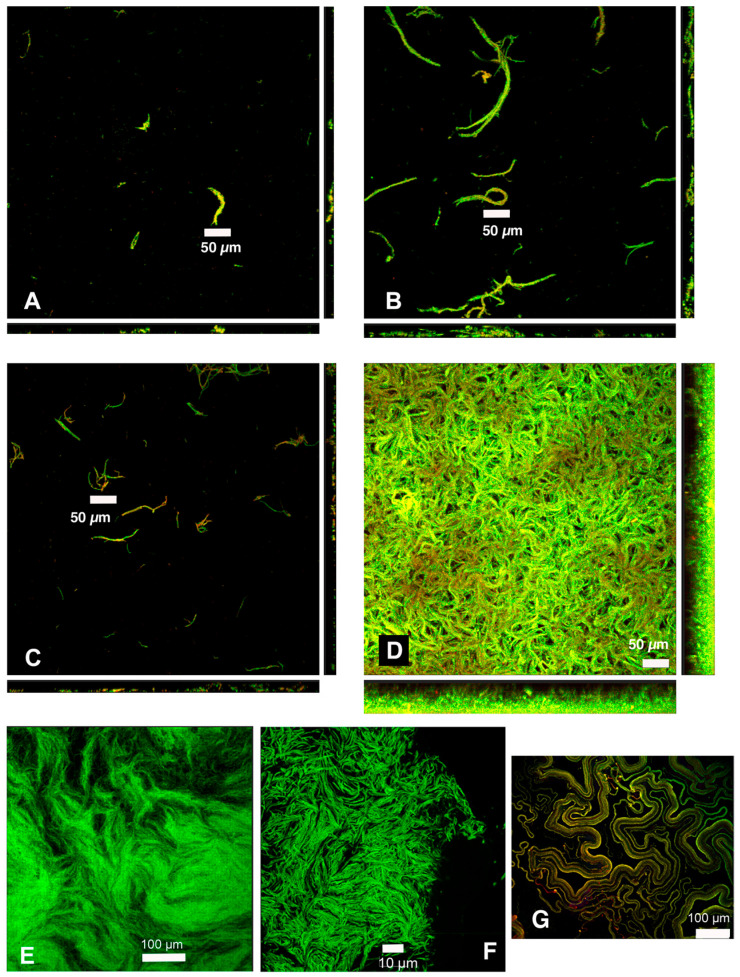
Confocal micrographs document cording in biofilms of *Mucispirillum schaedleri* ASF 457 and of *Mycobacterium* spp. (**A**) Chamber-slide-grown *M. schaedleri* biofilm after 18 h growth. Viability-stained. (**B**) Chamber-slide-grown biofilm after 48 h growth. (**C**) Flowcell-grown *M. schaedleri* biofilm after 18 h growth. Viability-stained. (**D**) Flowcell-grown biofilm after 48 h growth. Bar = 50 µm in (**A**–**D**). Rectangular panels beneath and to the right of the main (square) images in (**A**–**D**) are X-Z/Y-Z cross sections of the XYZ dataset in each main image. The thin biofilm in C has short cross sections, whereas the thick biofilm in (**D**) has tall cross sections. The height of the cross sections represents that of the entire biofilm (maximum-intensity projections of the entire dataset). (**E**,**F**) GFP-expressing *Mycobacterium bovis* pellicle biofilm from current study. (**G**) tdTomato-expressing *M. marinum* biofilm. Reprinted under license from reference [39].

**Figure 4:**
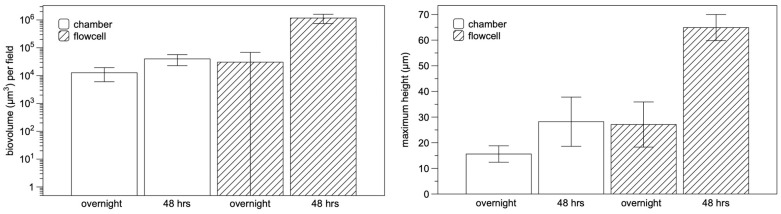
Comparison of chamber- and flowcell-grown *Mucispirillum schaedleri* biofilms. Biovolume (**left**) and maximum height (**right**) of chamber-grown and flowcell-grown *M. schaedleri* biofilms after 18 h and 48 h.

*M. schaedleri* ASF 457 presented a striking biofilm morphology. When grown in AMM/NO_3_ medium, it displayed large rope-like structures resembling masses of the closely appressed cells seen in planktonic broth culture; these structures will be termed cords. After 48 h of growth, cords were much larger than after 18 h of growth (Figure 3A–D), and surface coverage was much higher in the continuous culture system of flowcells than under static conditions in chamber slides. The biofilm morphology overall is the same as cording documented in mycobacterial biofilms, here shown in *M. bovis* biofilms grown as part of the present study (Figure 3E,F), as well as in *M. marinum* biofilms from different studies [38,39] (Figure 3G). Image analysis (Figure 4) demonstrated that the average biovolume per field of view in chambers increased 3-fold between 18 and 48 h (reaching 4.0 × 10^4^ µm^3^), and maximum biofilm height (measured as the height at which cells were no longer visible in the microscope) increased 1.8-fold (reaching 28.2 µm). In contrast, biovolume in flowcell-grown biofilms increased 40-fold (reaching 4.3 × 10^5^ µm^3^), and maximum height increased 2.4-fold (reaching 64.9 µm). At 18 h of growth, high standard deviation was apparent in average biovolume measurements for flowcell-grown biofilms, whereas the range of initial biovolumes in chamber-grown biofilms was comparatively narrow. However, by 48 h, flowcell-grown biofilms had reached confluence, whereas chamber biofilms still had uncolonized areas (Figure 3).

**Figure 5:**
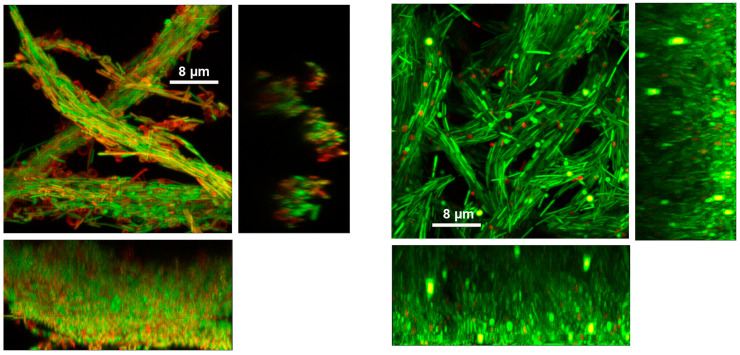
High-magnification confocal micrographs of viability-stained 48-hour-old chamber-grown *Mucispirillum schaedleri* biofilm (**left**) and flowcell-grown biofilm (**right**). Rectangular panels beneath and to the right of the main (square) image are side-on views. Supplementary Videos S1–S4 show the series of z-planes through the samples.

A feature common to ASF 457 biofilms regardless of growth condition was the presence of round bodies identical to those in broth cultures. Round bodies were more prominent and more likely to be stained PI-positive in chamber biofilms than in flowcell biofilms; round bodies in flowcell-grown biofilms often appeared completely green or red rather than containing a green or red cell along the inner wall (Figure 5 and Supplementary Materials—Videos S1–S4).

## 4. Discussion

The present study demonstrates three key findings: (1) *M. schaedleri* displays pleomorphic forms distinct from those reported for other gut bacteria, e.g., *Helicobacter* [40,41], but which are identical to round bodies described for spirochetes—these round bodies are seen in biofilms in vitro; (2) round bodies increase in *M. schaedleri* cultures in an age-dependent manner, and many round bodies are considered as “live” in a live/dead viability assay; (3) *M. schaedleri* biofilms in vitro display the distinct mycobacterial morphology known as cording.

In *T. denticola*, round bodies occur not only in broth culture, but also in biofilms in vitro [42] (an observation that has been documented here as well; Figure 1). They likely also occur in biofilms of *B. burgdorferi* [35] and may be a common feature among all spirochetes. The present study reports an age-dependent increase in round bodies in *M. schaedleri*, which is also a characteristic of treponeme round bodies.

*M. schaedleri* biofilms displayed a corded morphology strikingly similar to that described in *M. bovis* [43] (documented here as well; Figure 3) and in *M. marinum* [38,39]. In mycobacteria, genes known as cording factors are involved in mycolic acid synthesis/modification (e.g., mycolyltransferase antigen (Ag) 85 and phthiocerol dimycocerosate exporter mycobacterial membrane protein Large (MmpL7)) and in Type VII secretion (e.g., mycP, EsxA/B) [39,44]: both are important pathways in the production of extracellular polymeric substances (i.e., biofilm matrix). Cording in vitro is typically studied using mycobacterial biofilms grown as pellicles at the air–liquid interface; an exception is cording described in rotary cell culture [43]. In contrast to pellicle biofilms, submerged mycobacterial biofilms have a non-corded, lichen-like morphology [39]. Cording occurs intracellularly (i.e., within the host) and is thought to impair host immune surveillance [44]. Round bodies have not been described in mycobacteria. In contrast to mycobacteria, *M. schaedleri* is an anaerobic commensal, does not form biofilms at the gas–liquid interface and its genome does not contain cording factors [8]. Although the present study on *M. schaedleri* did not address the mechanism of cording nor its potential function in vivo, no cording or round bodies have been observed in sections of murine gut from gnotobiotic animals which have received rectal and oral gavages of *M. schaedleri* [8,18].

Viability staining demonstrated that in *M. schaedleri* biofilm cultures (particularly those formed in chamber slides), round bodies had reduced membrane integrity and/or transmembrane potential compared to rods, especially once the presence of an enclosed cell was difficult to verify. However, quantification of PI-stained round bodies in broth cultures demonstrated that many round bodies were viable, including at six days. The incorporation of the “dead” PI stain into bacterial cells depends on its ability to cross the cell membrane. In cells that have a high membrane potential (active cells) or intact membranes, the charged dye is excluded. However, the dye can enter cells that have damaged membranes. Thus, the observation that round bodies in chamber-slide biofilms take up PI to a greater extent than do round bodies from planktonic cultures suggests that the round bodies are less active (low transmembrane potential or damaged membrane). In addition, the round bodies that take up PI to the greatest extent also lack a clearly defined cell within. This suggests that these round bodies are damaged or never contained a cell in the first place.

Biofilm culture in chamber slides is a batch culture procedure. In contrast, culture in flowcells is analogous to a fixed-bed reactor in which fresh medium is constantly supplied; nutrient depletion can occur only if biomass and cellular activity become so high that nutrients are exhausted during transit through the flowcell. The present experiments document that sufficient nutrients were available to support growth of *M. schaedleri* biofilms in chambers over the period of 18–48 h, albeit with a small (3-fold) increase in biovolume. During this time, PI-positive round bodies became more prominent. However, in flowcells, growth over the same period resulted in a 40-fold increase in biovolume, and PI-positive round bodies were less apparent; instead, round bodies lacking a c-shaped cell but staining positive with Syto 9 were observable. The homogenous staining of round bodies suggests that these may contain the amorphous material seen in electron microscopy, but, in the case of Syto-positive cells, a membrane potential across the round body membrane remains. Such cells may also become PI-positive if nutrients are no longer plentiful. Results from biofilm experiments suggest that, once formed, round bodies may be more susceptible to senescence than are rods during extended periods of nutrient depletion.

Scant evidence exists for the presence of round bodies in spirochete infections. Likewise, cording is not easily identifiable in mycobacterial infections. A function for these forms has yet to be identified in vitro. The presence of both forms in *M. schaedleri* presents an opportunity to examine the relevance of round bodies and of cording in a tractable model system. Future directions should therefore include assessing whether round bodies appear in vivo and evaluating how *M. schaedleri* interacts with the host and the host’s indigenous microbiome using normal as well as gnotobiotic mouse models. These questions can be addressed using fluorescence in situ hybridization and RNA sequencing approaches.

## Data Availability

The method for identifying round bodies and rods based on *Eccentricity* and *Solidity* is shown in Supplementary Figure S1. Any additional microscopy images or extended biofilm movies will be made available upon request. MATLAB script used to identify and enumerate round bodies and rods by microscopy will be provided upon request.

## Supplementary Materials

The following supporting information can be downloaded at: https://www.mdpi.com/article/10.3390/microorganisms11092200/s1, Figure S1: Identification of round bodies and rods based on *Eccentricity* and *Solidity*; Table S1: Frequencies and viability of rods and round bodies in 3- and 6-day-old cultures; Video S1: Chamber 20X; Video S2: Chamber 100X; Video S3: Flowcell 20X; Video S4: Flowcell 100X.
