# Supplementary material for: Mucispirillum schaedleri: Biofilm Architecture and Age-Dependent Pleomorphy
Source: Microorganisms. 2023 Aug 31;11(9):2200. doi: 10.3390/microorganisms11092200 (PMC10535455; doi:10.3390/microorganisms11092200)
Supplement: Supplementary file 1 [file microorganisms-11-02200-s001.zip › Figure S1.pdf]

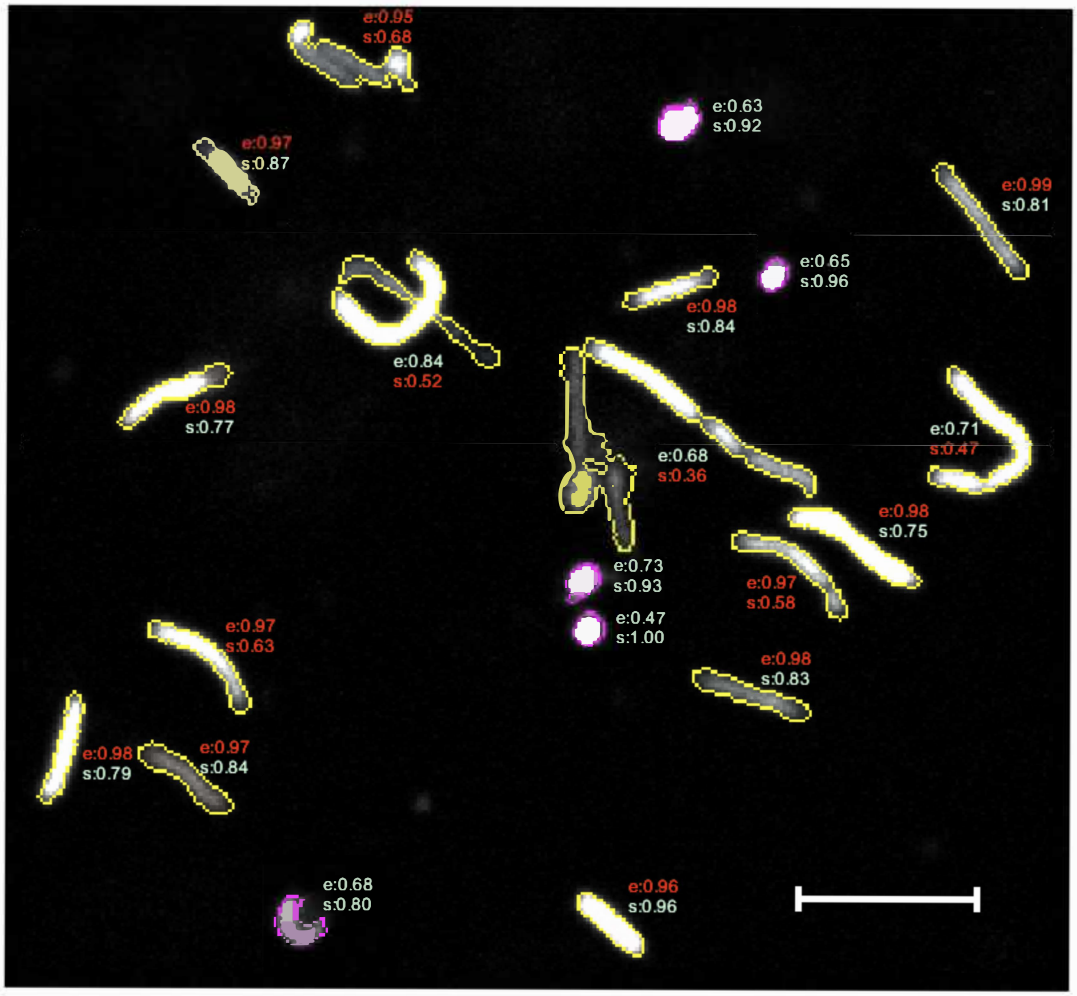

Supplementary Figure S1. Identification of round bodies and rods based on *Eccentricity* and *Solidity*. Objects outlined in fuchsia were identified as round bodies and objects outlined in yellow were identified as rods. *Eccentricity* (e) and *Solidity* (s) values are indicated beside each segmented object. A round-body corresponded to an *Eccentricity* value lower than 0.9 and a *Solidity* value greater than 0.7 (in white). All other values (in red) are associated with a rod-like shape. Bar=5µm.
