# Supplementary material for: Mucispirillum schaedleri: Biofilm Architecture and Age-Dependent Pleomorphy
Source: Microorganisms. 2023 Aug 31;11(9):2200. doi: 10.3390/microorganisms11092200 (PMC10535455; doi:10.3390/microorganisms11092200)
Supplement: Supplementary file 1 [file microorganisms-11-02200-s001.zip › Table S1.pdf]

Supplementary Table S1. Frequencies and viability of rods and round bodies in 3- and 6-day-old cultures.

Condition	**3-day-old culture**	**6-day-old culture**
**Live rods**		
Absolute count; mean (SD)	102 (22)	81 (27)
% (SD)	76.8 (4.3)	49.4 (12.7)
**Dead rods**		
Absolute count; mean (SD)	7 (4)	4 (5)
% (SD)	3 (2.9)	2.6 (1.6)
**Live round bodies**		
Absolute count; mean (SD)	37 (20)	43 (9)
% (SD)	15.3 (6.2)	36.5 (4)
**Dead round bodies**		
Absolute count; mean (SD)	11 (6)	20 (20)
% (SD)	5 (1.9)	11.5 (9.2)

SD = standard deviation. For each experimental condition per experiment, cell counts were obtained from 9 scanned images and averaged. Data represent the mean counts from 4 independent experiments.
